# Adolescents’ Personality Development – A Question of Psychosocial Stress

**DOI:** 10.3389/fpsyg.2021.785610

**Published:** 2021-12-17

**Authors:** Diana Raufelder, Frances Hoferichter, Stefan Kulakow, Sabrina Golde, Tobias Gleich, Lydia Romund, Robert C. Lorenz, Patricia Pelz, Anne Beck

**Affiliations:** ^1^Institute of Educational Science, University of Greifswald, Greifswald, Germany; ^2^Department of Education and Psychology, Free University of Berlin, Berlin, Germany; ^3^Clinic for Psychiatry and Psychotherapy, Charité – Universitätsmedizin Berlin, Berlin, Germany; ^4^Max Planck Institute for Human Development, Berlin, Germany; ^5^HMU Health and Medical University Potsdam, University of Potsdam, Potsdam, Germany

**Keywords:** stress, personality, adolescence, fMRI, psychosocial stress

## Abstract

Following the relational-developmental systems approach, this three-wave study examines whether acute stress (T2) mediates the relationship between the development of personality traits from the beginning of 8th grade (T1, *M*_*age*_ = 15.63, *SD* = 0.59; 22 girls) to the end of 9th grade (T3). Using the Montréal Imaging Stress Task, which is a task that provokes acute social stress by negative social feedback, this study combined the functional magnetic resonance imaging (fMRI), heart rate, and longitudinal survey data of 41 adolescents. Mediation analysis revealed that stress-induced left insula activation partially mediates the longitudinal stability of conscientiousness. These results highlight the impact of negative social feedback during stress on students’ personality development.

## Introduction

As children develop within a social frame of relationships, cognitive and behavioral developmental changes may be particularly accompanied by social stressors. Main sources of adolescents’ social stress are problems and troubles with parents, peers, teachers and school-related pressures (Murberg and Bru, 2004; Byrne et al., 2007), which might be shaped by the changes in the brain’s socio-emotional system (Steinberg, 2008) with the onset of puberty. At the same time, these social environments are crucial for adolescents’ personality development, as they contribute to an adolescent’s sense of “who I am.” Considering these distinctive developmental changes that are embedded within social relationships and the brain, it is not surprising that adolescence defines a critical period for the personality development of young adults. Hence, a crucial developmental challenge during adolescence may be the processing of stressors and learning from social feedback.

According to the relational-developmental systems perspective (Lerner and Lerner, 2019), the formation of identity and personality is constantly and reciprocally shaped through social interactions, including the social feedback from the environment that children and adolescents grow up in. Particularly during adolescence, the brain undergoes architectural changes as the developing brain is highly susceptible to environmental stimuli and as such is vulnerable to stressors (Tottenham and Galvan, 2016). While personality traits build the core of the self, identity formation is the core developmental task during adolescence. With gaining the cognitive capacities to engage in abstract thinking, adolescents form an identity, which provides meaning to life and goes along with profound changes in personality traits (Klimstra, 2013). This developmental process is mainly influenced through social environments, as the way adolescents see themselves changes in response to peers, family, and school. To state this in more detail, environmental stimuli trigger inner biological and physiological processes (e.g., brain activity), which in turn are integrated in children’s personalities and vice versa. Yet, it is this interaction that determines how environmental stimuli, such as social feedback and stressors, are perceived and cognitively processed (Fishman et al., 2011; Childs et al., 2014).

While there is a growing body of research highlighting the dramatic changes related to social cognition during adolescence (Blakemore, 2008; Burnett et al., 2011), as well as how perceived stress may interact with the architecture of the developing brain (Lupien et al., 2009), there remains a research gap with regard to how personality development from early to middle adolescence is mediated by stress reactivity to negative social feedback. The present study has been conceptualized to address this research gap, using longitudinal survey data and the fMRI data of 41 adolescent students.

### The Big Five and Their Relevance for Future Life Outcomes

Personality research is largely dominated by the five-factor model (FFM) and has become a popular model for assessing personality due to its cross-cultural validity (McCrae and Costa, 1997, 2008). These five factors are usually labeled *emotional instability, extraversion, autonomy, agreeableness*, and *conscientiousness*, although these labels occasionally differ across studies and instruments (Hendriks et al., 1999). In the FFM, the trait *emotional instability* refers to experiencing negative emotions, such as anxiety, irritation, sadness, and insecurity, in a frequent and intense manner (Shiner, 2019). Emotional instability is linked to various negative and maladaptive outcomes, such as attentional difficulties and problems related to cognitive processing (Shackman et al., 2016). Moreover, particularly in stressful situations, emotional instability may thwart motivation and enable inaction and avoidance (Connor-Smith and Flachsbart, 2007). *Extraversion* relates to the personality trait that is associated with talkativeness, boldness, sociability, enthusiasm, and positive emotionality (Smillie et al., 2019). However, Lucas et al. (2000) argue that the core of extraversion is not sociability but reward sensitivity, while Ashton et al. (2002), p. 245) describe extraversion as a tendency to “behave in ways that attract social attention.” Extraversion is linked to numerous – generally positive – outcomes, such as a more active social life and closer relationships (Lucas et al., 2008) and general well-being (Sun et al., 2018). *Openness to experience* (autonomy) refers to curiosity and cognitive exploration in terms of gathering and engaging with new information (DeYoung, 2015; Schwaba, 2019). It is further linked with active strategies in coping with stress (Malkiewicz, 2014). The major correlates of openness to experience are creativity (Kandler et al., 2016), intelligence (DeYoung et al., 2012), and academic achievement (Noftle and Robins, 2007). *Agreeableness* encompasses characteristics such as compassion, empathy, compliance, politeness, and modesty (Soto and John, 2017; Tackett et al., 2019). Agreeableness is negatively associated with externalizing problems, such as antisocial behavior (Decuyper et al., 2009). Particularly with regard to children and adolescence, some studies have shown that agreeableness is also associated with internalizing problems, and can be seen in social withdrawal, anxiety, and depressive symptoms (De Clercq et al., 2008; Laursen et al., 2010). Moreover, agreeableness appears to be associated with various aspects of the quality of relationships. For example, studies have found associations with better peer relations (Laursen et al., 2010) and higher peer closeness (Neyer and Lehnart, 2007). Finally, *conscientiousness* refers to “the propensity to be self-controlled, responsible to others, hardworking, orderly and rule abiding” (Jackson and Roberts, 2017, p. 133). The trait of conscientiousness is often related to self-oriented perfectionism (Stoeber et al., 2009), effortful control (e.g., sustained attention, persistence, planning) in the case of children and adolescents (Jackson and Hill, 2019), and generally to numerous future life outcomes, such as health and longevity (Jackson et al., 2015) and academic achievement (Noftle and Robins, 2007).

Personality traits and stress susceptibility have been shown to be intertwined in complex ways. In a study that investigated the response to acute stress induced by the Trier Social Stress Test (TSST) – a commonly used psychosocial stress task – in relation to multidimensional personality traits, Childs et al. (2014) found that adults with high negative emotionality showed greater aversive mood and lower blood pressure. In turn, individuals with high agentic positive emotionality (i.e., reward sensitive individuals with a tendency toward social dominance and assertiveness) exhibited prolonged heart rate responses to stress. Individuals with high communal positive emotionality (i.e., social warmth and affiliation) exhibited lower cortisol and blood pressure responses. The authors argue that a lack of positive feedback during the stress task means that reward sensitive individuals may lack a sense of achievement, which causes a prolonged stress reaction, while individuals with close interpersonal relationships may be more stress-resilient (Papousek et al., 2011; Childs et al., 2014). Similarly, Xin et al. (2017) found lower heart rate, diminished cortisol levels, lower positive affect, and controllability in response to the TSST among individuals with high emotional instability. These findings indicate a down regulation within the autonomic nervous system and hypothalamic-pituitary-adrenal system among individuals with high trait emotional instability, who generally tend to experience higher levels of stress, anxiety, and affective instability (Shiner, 2019). The investigation by Xin et al. (2017) also revealed that individuals scoring high on extraversion and openness exhibited less cortisol activation after being exposed to the TSST, and that extraverted individuals showed less increase in negative effect. Numerous studies have been conducted to investigate personality-brain mechanisms. They have investigated the brain functional organization of healthy adolescents during resting state brain activity, i.e., during the absence of an explicit task within the scanner. Although these studies reveal that personality traits are distinctively related to brain networks, the results are mixed and do not provide a full understanding of how personality relates to the architecture of the brain (Li et al., 2017; Mulders et al., 2018).

### Personality Development Through Adolescence

The FFM has become a popular model for assessing personality due to its cross-cultural validity (McCrae and Costa, 1997) and its high stability across large age spans. However, in contrast to adults, adolescents show notably less stability in the five factors, indicating that personality features are subject to change during that time (McCrae et al., 2002). While different studies indicate certain trends, there is a lack of overall consistency across studies (Göllner et al., 2017). Theorists of FFM predominantly explain these inconsistencies by the fact that, with the onset of adolescence, individuals start to reflect about themselves, their interactions with others, and how they may be perceived by others (see Golde et al., 2019). In addition to fluctuations in the personality of youths, another challenge is related to the psychometric properties of the instruments used to assess the personalities of children and adolescents. Often, these instruments exhibit reliabilities that vary greatly depending on the age of students. Prior studies could indicate that particularly in late childhood and early adolescence, researchers occasionally face alpha values of below 0.40 values. However, there is agreement that until late adolescence and early adulthood these fluctuations in reliability level out and the constructs become more stable (Soto et al., 2011; Göllner et al., 2017). Besides these reliability concerns, other studies have shown that such scales can be used nonetheless and are still valid (Gosling et al., 2003; Rammstedt and John, 2007). Furnham (2008) for example highlighted the role of a small number of items used for scale construction which often causes considerate drops in reliability.

### The Role of Stress in Adolescents’ Brain Activity

A reason for the low stability of personality traits throughout adolescence might lie in the biological development during this period. In fact, adolescence has been identified as a sensitive period, which is accompanied by structural and functional changes in the brain (Blakemore, 2008; Fox et al., 2010; Tottenham and Galvan, 2016; Braams and Crone, 2017; Guyer et al., 2018).

Neuroimaging studies have identified several brain regions associated with social stressors, including the insular cortex and the middle temporal cortex (Blakemore, 2008; Wang et al., 2017). The right insula is an index of social distress that is usually involved in social pain, and is associated with the processing of social rejection (Eisenberger et al., 2003; Kross et al., 2007; Kashdan et al., 2014). Previous studies have shown that low-trait resilient participants exhibited greater insula psychosocial stressors than high-trait resilient participants (Waugh et al., 2008; Shao et al., 2018). Insula activation is also associated with high anxiety (Chua et al., 1999; Simmons et al., 2006). Moreover, Slavich et al. (2010) were able to show that the activation of the bilateral insula during social exclusion was associated with inflammatory response. In addition, the insula has been implicated in various affective and emotional processes, as well as interoceptive processes (Critchley, 2005; Craig, 2009). In a study on emotional intelligence (EI), i.e., the ability to identify, regulate, and process the emotions of oneself and others, Quarto et al. (2016) found that EI scores were related to left insula activation during the social judgment of emotional faces. Jabbi et al. (2008) argue that insula activation can be described as a mirror, neuron-like link between external and internal experiences.

During brain related, stress-associated research, the middle temporal cortex was often shown to be involved. It is said to be a multimodal association site that encompasses the integration of memory, audiovisual information, and object recognition functions (Calvert, 2001; Gogtay et al., 2004). In addition to its relationship to episodic and semantic memory (Price, 2010), there are also indices that suggest that the middle temporal gyrus plays an important role within a large-scale network related to executively demanding goal-oriented cognition (Duncan, 2010; Davey et al., 2016). The middle temporal regions are also associated with creative insight, that is, with finding creative ways to solve complex problems (Shen et al., 2017). By contrast, temporal gyrus activity appears to be inhibited during experiences of stressors, as Pruessner et al. (2008) found decreased neural activity of the temporal gyrus and other regions in the limbic system of adults when inducing psychosocial stressors during PET and fMRI. These decreases in activation were accompanied by increases of the endocrine stress marker cortisol. While Pruessner et al. (2008) argue that these areas constantly “scan” the environment for potential threats and “shut down” as a consequence of threat, Golde et al. (2021) assume that these functions are still developing during adolescence. This argument is supported by the fact that the middle temporal cortex undergoes substantial maturation during the first decades of people’s lives, reaching a peak of full maturation at approximately 16 years old (Blakemore, 2008). While DeWall et al. (2011) argue that the need to belong, that is, to feel socially included, is a core personality trait that all psychologically healthy humans share, independent of their personality traits, we investigate how the big five personality traits of young adolescents determine the activation of selected brain regions during a stress task with negative social feedback.

### Aims and Hypotheses

Based on the relational-developmental systems model (Lerner and Lerner, 2019) and the empirical findings discussed above, the following hypotheses were tested:

Hypothesis 1: Big five and insula activity are significantly associated during an event with task-induced stress (see below for Montréal Imaging Stress Task; MIST) with negative feedback:
H1.1: As the insula cortex has been shown to be related to social pain and rejection, we expect students with high agreeableness at the beginning of 8th grade, that is, students characterized by cooperativeness, warmth, and consideration for others, to exhibit greater insula activation at the beginning of 9th grade than students who score low on agreeableness.
H1.2: Similarly, we expect students who score high on extraversion at the beginning of 8th grade, that is, students who strive for social attention and are sensitive toward social reward, to show higher insula activation at the beginning of 9th grade in comparison to students with low extraversion.
H1.3: As low-trait resilient individuals have shown to exhibit greater insula responses to psychosocial stressors, we expect students with low emotional stability at the beginning of the 8th grade to exhibit greater insula activity at the beginning of 9th grade.
H1.4: We further expect insula activity to mediate the relationships between agreeableness, extraversion, and emotional instability from the beginning of 8th grade to the end of 9th grade, respectively.

Hypothesis 2: Big five and middle temporal gyrus activity are significantly associated during an event with task-induced stress with negative feedback:
H2.1: As the middle temporal gyrus is associated with executively demanding goal-oriented cognition, we expect conscientious students at the beginning of 8th grade, that is, students who are orderly, self-controlled, and hardworking, to exhibit higher middle temporal gyrus activity at the beginning of 9th grade than students scoring low on conscientiousness.
H2.2: As middle temporal activity is also related to creative insight and problem solving of complex challenges, which is inhibited during experiences of psychosocial stress, we expect that students who score high on autonomy (i.e., who are curious, creative, and who cognitively explore new information, which is also referred to as openness) at the beginning of 8th grade to exhibit lower middle temporal activity at the beginning of 9th grade.
H2.3: We also expect middle temporal activity to mediate the development of students’ conscientiousness, as well as their autonomy, during the course of their school years.

Hypothesis 3: Big five and heart rate during a stress induced event with negative feedback:
H3.1: As previous studies indicate low neuroendocrine activation (e.g., heart rate, blood pressure, cortisol level) among individuals scoring high on emotional instability during induced stress events (Childs et al., 2014; Xin et al., 2017), we expect students with high emotional instability at the beginning of 8th grade to exhibit lower heart rates at the beginning of 9th grade.
H3.2: We also expect low heart rates among students scoring high on agreeableness at the beginning of grade eight, as they tend to have high quality relationships (Neyer and Lehnart, 2007; Laursen et al., 2010) that protect them from experiencing stressors (Papousek et al., 2011; Childs et al., 2014; Hoferichter et al., 2014).
H3.3: We also expect conscious students at the beginning of grade eight to exhibit higher heart rates at the beginning of 9th grade, as the negative feedback of not sufficiently being able to fulfill the calculation task (MIST) may induce stress in individuals scoring high on self-control, orderliness, and perfectionism (Stoeber et al., 2009).

## Materials and Methods

### Participants and Procedure

This study is part of a larger two-wave study with secondary school students from Brandenburg, Germany (SELF-project; *N* = 1088 at Time 1, *M_*age*_* = 13.7 years; *SD* = 0.53), which was approved by the Brandenburg Department of Education, Youth, and Sports. The questionnaire data used in this study were collected in class at the beginning of 8th grade (Time 1: T1) and the end of 9th grade (Time 3: T3). At least two research assistants were present during the research, who presented aims and procedure of the study, explained the use of Likert scales and answered any questions students had during the process of the study. Before the data collection, written permission was obtained from the participating schools, parents, and students.

After the first questionnaire-based data collection, a total of 53 adolescents were randomly selected from this larger sample pool. The students were invited to take part in a fMRI study (Time 2: T2), which lasted about 45 min. Students were offered a compensation of 100€. The fMRI study was additionally approved by the ethics committee of the German Psychological Society. Before the fMRI session, which took place in the beginning of 9th grade (T2), all participants as well as legal guardians provided written informed consent. All students were informed that they can cancel their participation at any time. Before the fMRI session, in which the participants performed a psychosocial stress task, standardized questionnaires were used to control for the following exclusion criteria: (1) adverse health conditions, (2) neurological or mental disorders, (3) use of medication that influences central nervous system, (4) non-removable ferromagnetic material. During the fMRI task, pulse oximetry was used to record heart rate. Overall, 10 participants had to be excluded from the fMRI sample due to excessive head movement (>3° mm translation or 3° rotation) and two participants due to technical problems during fMRI recordings. Finally, the following analyses are based on a sample of *N* = 41 adolescents (*M*_*age*_ = 15.63, *SD* = 0.59, 22 girls); one left-handed and 40 right-handed (according to the Edinburgh Handedness Inventory).

### Questionnaire Measures

#### Personality

Personality was measured with the Big Five Model of Personality scale (FFPI-J) for adolescents (Szirmak, 2005) based on the FFPI for adults (Hendriks et al., 1999). Participants rated statements on a 5-point Likert scale from 1 (“does not apply at all”) to 5 (“fully applies”). The FFPI-J consists of five subscales with five items each: The subscale emotional instability showed a good reliability (T1: α = 0.78; T3: α = 0.80) (e.g., “I have a negative feeling about the future”). The subscale autonomy, showed a low reliability (T1: α = 0.62; T3: α = 0.62) (e.g., “I know, what I want”). The subscale extraversion showed a low reliability at T1, but a satisfying reliability at T3 (T1: α = 0.66; T3: α = 0.70) (e.g., “I make an effort for others”). The subscale agreeableness also showed a low reliability at T1, but a satisfying reliability at T3 (T1: α = 0.64; T3: α = 0.74) [e.g., “I use others for my purposes” (recoded)]. The subscale conscientiousness showed an acceptable reliability (T1: α = 0.70; T3: α = 0.70) (e.g., “I like to have a regular day-to-day routine”).

### Neuroimaging Procedure

#### The Montreal Imaging Stress Task

To assess adolescents’ psychosocial stress reaction, a modified version (Zschucke et al., 2015) MIST (Dedovic et al., 2005; Pruessner et al., 2008) was performed in the fMRI scanner. The MIST contains a series of computerized mental arithmetic challenges, which change in terms of time limit and difficulty in order to increase the failure rates of the participants. These arithmetic challenges are accompanied by social evaluative threat components, such as negative feedback from the examiner.

**FIGURE 1:**
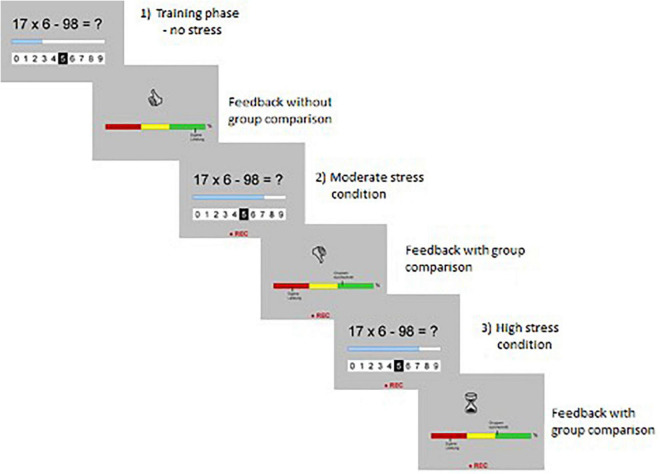
Process of the modified Montréal Stress Imaging Task (MIST) (Zschucke et al., 2015).

More specifically, the modified MIST (Zschucke et al., 2015) consists of three conditions: (1) low stress, (2) moderate stress and (3) high stress, which are performed in a successive order. While the participant lies in the scanner, he/she looks at a mirror above the head coil to reflect the screen, which shows a mental arithmetic task, a horizontal line with integers ranging from 0 to 9 to solve the task and a time bar, which shows the participant how much time is left for the entire trial (see Figure 1).

The participant can rate and submit the answer with a fiber-optic light sensitive button box, which navigates the cursor of the horizontal line from left to right and vice versa. First, the low stress condition consists of 40 trials with 20 s to solve each arithmetic task. Through the microphone, the examiner informs the participant that these trials are a training session. The results of these 40 trials are used to estimate participants’ individual baseline response time. Second, the moderate stress condition consists of 32 trials with 25% less time than participants’ baseline response time. This time, the examiner informs the participant that the results will now be recorded and monitored. In addition, two performance indicators appear on the screen: one indicator displays the participants’ performance, while the other shows a faked average performance of the other study participants. The latter is calculated in a way that it exceeds the present participant’s performance throughout the whole session. Subsequently, the examiner gives scripted negative feedback by using the microphone. To induce stress, the examiner asks the participant to make more of an effort to improve the performance for the last session, otherwise the data could not be used for the study (which is not true). Third, the high stress condition consists of 32 trials with further restricted time and higher task difficulty, so that the individual failure rate is approximately 50%. During the high stress condition, no feedback is given by the examiner. After completion of the task, participants were informed that it was impossible to accomplish the tasks correctly within the given time and that the test does not indicate their math abilities at all.

The MIST has been shown to induce significant psychoendocrine stress reaction in numerous previous studies (see Dedovic et al., 2005; Pruessner et al., 2008). Continuous heart rate was recorded by pulse oximetry over the course of the task and mean values for each condition (low, moderate, high) were calculated.

fMRI data were acquired using a 3 Tesla Magnetom Trio scanner system (Siemens Medical Systems, Erlangen, Germany) with a 12-channel radiofrequency head coil at the XXXX, Germany. Details on imaging acquisition and preprocessing of imaging data can be found in Supplementary Document A.

### Data Analyses

#### Single Subject Analysis

In order to eliminate low-frequency components (1/256 Hz cut-off frequency), a high-pass filter was applied for the time series. Initially, a 1st order autoregressive model with aliasing effects and serial correlations was computed with restricted maximum likelihood (ReML) algorithm in SPM. Subsequently, a general linear model (GLM) was conceptualized at the single-subject level (see Zschucke et al., 2015). The different conditions were estimated convolved with a hemodynamic response function as explanatory variables on a voxel-by-voxel basis. The main neural parameter, which represents the stress-related neural activity of each condition, the mean signal within each run (constant of the GLM) was used. As regressors of no interest, motor response, task and feedback screen, as well as the six movement parameters were additionally implemented. For both, the sustained effects of each stress level (i.e., baseline contrast images) and the effects of the comparison between high and low stress levels (i.e., [high > low], [low > high]), contrast images of parameter estimates were assessed (see Golde et al., 2021).

#### fMRI Data Analyses

**FIGURE 2:**
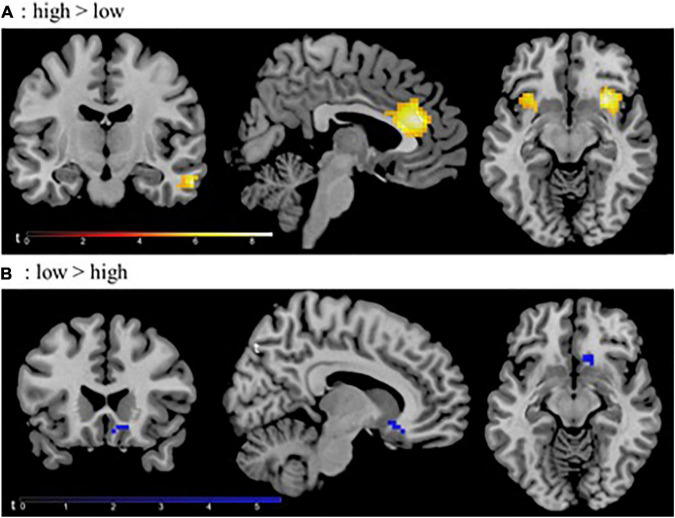
Significant activation clusters of one-sample *t*-test thresholded at *p* < 0.05 FEW-corrected for the whole brain; **(A)** contrast high > low stress condition, **(B)** contrast low > high stress condition; L, left hemisphere.

One sample *t-*tests with differential contrast images [low vs. high] from individual subjects were computed with a threshold of *p* < 0.05 whole brain family wise error (FWE)-corrected, with a minimum cluster size of *k* ≥ 5 voxels. This resulted in significant clusters of increasing stress-associated neural activation during high > low stress condition in the anterior cingulate gyrus (ACC), right and left insula, right middle temporal gyrus and the angular gyrus (see Figure 2). Significant decreasing activation from high stress to low stress was found in the subcallosal gyrus. Details can be found in Supplementary Table A. From all these significant activation clusters mean parameter estimates for the whole cluster were extracted for each participant for further analysis in Mplus.

#### Multiple Regression Analysis With Indirect Effects

Using Mplus Version 8.1 (Mutheén and Mutheén, 1998–2013) multiple regression analysis (MRA) was performed with robust maximum likelihood (MLR) estimator, which is robust against violations of normality assumptions (Yuan and Bentler, 2000). Indirect effects were computed to assess the potential mediating effects from each personality factor at T1 to the same factor at T3 via stress-induced neural activity (T2) by constructing confidence intervals around the estimates (MacKinnon, 2008; Preacher and Hayes, 2008). This procedure reduces the bias caused by the non-normality in the sampling distribution of indirect effects (Shrout and Bolger, 2002). Model fit was estimated using the following fit indices, as suggested by several authors (West et al., 2012): Chi-square test of model fit (χ^2^), root mean square error of approximation (RMSEA), comparative fit index (CFI), and standardized root mean square residuals (SRMR). An acceptable fit to the data is usually indicated with CFI values greater than 0.90, RMSEA and SRMR values lower than 0.08. Full information maximum likelihood (FIML) procedure was used to handle missing data. This procedure is unbiased under the missing at random (MAR) assumption, and retains statistical power, because no observations are deleted (Enders, 2010). Additionally, heart rate was added as a covariate in order to improve maximum likelihood estimation under the MAR assumption (Graham, 2003).

## Results

### Descriptive Statistics and Correlations

Before the regression analysis bivariate correlations with all identified brain region activations during MIST (see above) and the big five personality components have been conducted. However, only the right insula (RINS), the left insula (LINS) and the right middle temporal gyrus (RMIDTEMP) as well as the heart rate during high > low stress condition were significantly associated with the big five personality factors (Table 1). Accordingly, the following analyses are based on these variables. The manifest descriptive statistics for each variable (range, mean, standard deviation, skewness, kurtosis) are displayed in Table 1.

**TABLE 1:** Intercorrelations between variables of interest, mean, standard deviation (SD), range, skewness and kurtosis.

Measure	2	3	4	5	6	7	8	9	10	11	12	13	14	*M*	*SD*	Range	Skewness	Kurtosis
(1) EmIn T1	0.11	0.09	–0.21	0.38**	0.46**	–0.00	0.17	0.05	0.20	0.37*	0.17	0.16	–0.17	2.37	0.49	1–5	0.32	0.05
(2) Auto T1	–	0.44***	−0.31*	0.39**	–0.16	0.67***	0.37**	–0.28	0.24	–0.13	–0.06	–0.17	0.10	3.67	0.27	1–5	–0.12	0.07
(3) Extra T1		–	–0.06	0.32*	0.01	0.35**	0.83***	0.20	0.30	0.12	0.25*	–0.22	–0.14	3.39	0.48	1–5	–0.05	–0.49
(4) Agree T1			–	–0.17	–0.14	−0.32*	0.02	0.59***	–0.22	0.23*	0.22*	0.32*	−0.45**	3.73	0.45	1–5	–0.25	–0.48
(5) Cons T1				–	–0.20	0.53***	0.28	0.16	0.51**	0.32*	0.37**	–0.23	0.27	2.89	0.48	1–5	0.44	0.07
(6) EmIn T2					–	–0.27	0.06	–0.25	0.02	0.06	0.02	0.20	–0.14	2.45	0.49	1–5	–0.08	–0.25
(7) Auto T2						–	0.27	–0.13	0.27	–0.01	0.10	−0.46**	0.11	3.63	0.30	1–5	0.07	–1.01
(8) Extra T2							–	0.18	0.16	0.09	0.20	–0.12	–0.14	3.49	0.47	1–5	0.09	–0.79
(9) Agree T2								–	–0.13	0.33	0.24	–0.04	–0.11	3.57	0.54	1–5	–0.25	–0.36
(10) Cons T2									–	0.23	0.41**	–0.09	0.18	3.04	0.39	1–5	0.18	–0.55
(11) Rins										–	0.79***	0.34***	0.18	1.98	2.49	–	0.00	3.66
(12) Lins											–	0.11	0.24*	2.24	4.88	–	0.28	0.90
(13) Rmidtemp												–	–0.12	1.43	1.93	–	0.30	0.49
(14) Heart rate													–	72.66	313.18	–	0.70	0.38

*The reported coefficients are standardized.*

*p < 0.05, **p < 0.01, ***p < 0.001.

*Brain activation refers to contrast high > low stress.*

*EmIn, emotional instability; Auto, autonomy; Extra, extraversion; Agree, agreeableness; Cons, conscientiousness; Rins, right insula; Lins, left insula; Rmidtemp, right middle temporal gyrus; T1, Time 1 (beginning of 8th grade); T2, Time 2 (end of 9th grade).*

### Multiple Regression Analysis With Indirect Effects

**FIGURE 3:**
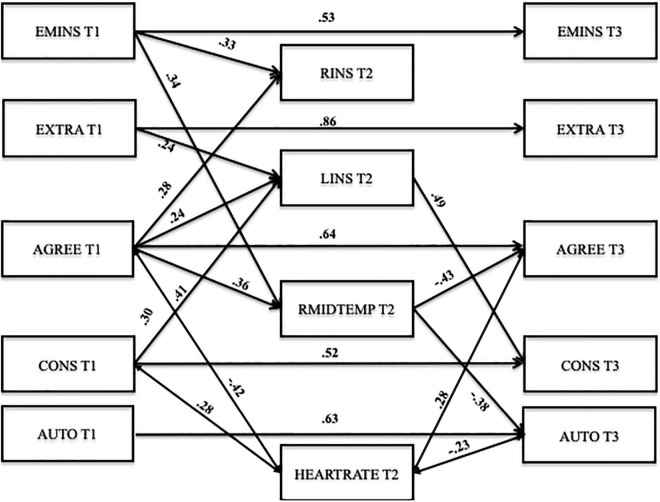
Multiple regression analysis (MRA) with indirect effects. Significant effects (*p* < 0.05) shown as standardized coefficients (β). For reasons of clarity the non-significant paths are not included in the figure. The covariance between the predictor, mediator and outcome variables are not included in the figure but explained in the manuscript. Emins, emotional instability; Extra, extraversion; Agree, agreeableness; Cons, conscientiousness; Auto, autonomy; Rins, right insula; Lins, left insula; Rmidtemp, right middle temporal gyrus; T1, Time 1 (beginning of 8th grade); T2, Time 2; T3, Time 3 (end of 9th grade); brain activation refers to contrast high > low stress.

A multiple regression analysis with indirect effects with all five personality factors and the neural activity of the RINS, LINS, and RMIDTEMP was computed. The heart rate of high stress condition was included as a control variable and as such correlated with each variable in the model. This model showed an acceptable fit: χ^2^(20) = 26.59, *p* > 0.05, CFI = 0.97, RMSEA = 0.09 (90% CI 0.00–0.17), SRMR = 0.08 (see Figure 3). The RMSEA is a bit high, but in “models with small *df* and small sample size, the RMSEA too often falsely indicates a poor fitting model. We recommend not computing the RMSEA for small *df* models, especially those with small sample sizes” (Kenny et al., 2015, p. 486).

#### Direct Effects

All autoregressive paths between each personality factor at T1 with the same personality factor at T3 were found to be significant, which indicates stable personality factors from early to middle adolescence: emotional instability (*B* = 0.55, β = 0.53, *SE* = 0.15, *p* < 0.01), extraversion (*B* = 0.84, β = 0.86, *SE* = 0.06, *p* < 0.001), agreeableness (*B* = 0.68, β = 0.64, *SE* = 0.09, *p* < 0.001), conscientiousness (*B* = 0.49, β = 0.52, *SE* = 0.11, *p* < 0.01), and autonomy (*B* = 0.68, β = 0.63, *SE* = 0.09, *p* < 0.01).

Agreeableness at T1 positively predicted the increase of neural activity of the RMIDTEMP (*B* = 0.73, β = 0.36, *SE* = 0.13, *p* < 0.01), the RINS (*B* = 0.66, β = 0.28, *SE* = 0.13, *p* < 0.05), and the LINS (*B* = 0.79, β = 0.24, *SE* = 0.10, *p* < 0.05) from low to high stress condition. Emotional instability at T1 positively predicted the increase of neural activity of the RMIDTEMP (*B* = 0.68, β = 0.34, *SE* = 0.14, *p* < 0.05) and the RINS (*B* = 0.75, β = 0.33, *SE* = 0.12, *p* < 0.01) from low to high stress condition. In addition, conscientiousness at T1 (*B* = 1.31, β = 0.41, *SE* = 0.12, *p* < 0.001) and extraversion at T1 (*B* = 0.78, β = 0.24, *SE* = 0.10, *p* < 0.05) positively predicted the increase of neural activity of LINS from low to high stress condition.

Furthermore, the neural activity of the MIDTEMP negatively predicted agreeableness at T3 (*B* = –0.22, β = –0.43, *SE* = 0.14, *p* < 0.01) and negatively predicted autonomy at T3 (*B* = –0.15, β = –0.38, *SE* = 0.12, *p* < 0.01). The LINS positively predicted conscientiousness at T3 (*B* = 0.14, β = 0.49, *SE* = 0.22, *p* < 0.05).

#### Indirect Effects

In total, only one indirect effect was found to be significant: The neural activity in the LINS partially mediated the longitudinal stability of conscientiousness (*B* = 0.19, β = 0.20, *SE* = 0.09, 95% CIs [0.02,0.38]).

#### Covariance

Conscientiousness at T1 was positively associated with emotional instability at T1 (*r* = 0.38, *p* < 0.01), extraversion at T1 (*r* = 0.32, *p* < 0.05), and heart rate (*r* = 0.28, *p* < 0.05). The heart rate was negatively associated with agreeableness at T1 (*r* = –0.42, *p* < 0.001). Interestingly, conscientiousness at T3 was negatively related to extraversion at T3 (*r* = –0.22, *p* < 0.05). Emotional instability at T3 was negatively associated with agreeableness at T3 (*r* = –0.29, *p* < 0.05). Autonomy at T3 was negatively associated with heart rate (*r* = –0.23, *p* < 0.05), whereas agreeableness at T3 was positively associated with heart rate (*r* = 0.28, *p* < 0.01). Furthermore, the neural activity of the RINS was positively associated with the MIDTEMP (*r* = 0.36, *p* < 0.01), the LINS (*r* = 0.75, *p* < 0.001), and heart rate (*r* = 0.39, *p* < 0.001). The LINS was also positively related to heart rate (*r* = 0.37, *p* < 0.001). None of the other covariance were significant.

## Discussion

The current study investigated the development of personality traits of secondary school students over two school years, that is, from the beginning of eighth grade to the end of ninth grade, mediated by their psychosocial stress reaction during the MIST at the beginning of ninth grade.

More specifically, correlation analysis revealed that students with high agreeableness, conscientiousness, and emotional instability at the beginning of 8th grade showed higher neural activity of the right insula during the social stress task in comparison to students who scored low on these personality traits. By contrast, left insula activation was related to students’ extraversion, agreeableness, and conscientiousness at the beginning of 8th grade, and to conscientiousness in 9th grade.

Similarly, path analysis revealed that students with high agreeableness, extraversion, and emotional instability in 8th grade exhibited greater insula activity, which is in line with H1.1. More specifically, agreeable students who report friendly and helpful behavior, strive for social harmony, and are willing to put aside their own interests for the sake of others, appear to be stimulated by negative social feedback associated with a responsive insular cortex. As the insular cortex is associated with social pain, rejection, and emotional intelligence (Eisenberger et al., 2003; Kross et al., 2007; Kashdan et al., 2014; Quarto et al., 2016), one could argue that students with high agreeableness are neurobiologically challenged by being rejected. In detail, these students may perceive negative social feedback as more disturbing than others due to their pronounced need for social harmony. As the social harmony is disturbed by negative environmental stimuli, students expend resources in order to restore and maintain social harmony. The spending of resources maps directly to neurobiological processes that are triggered to regain the desired harmony. As expected (H1.3), students who reported emotional instability at the beginning of 8th grade exhibited greater (right) insula activity at the beginning of 9th grade, which is in line with previous research, and indicates that low-trait individuals show greater insula response to psychosocial stressors (Waugh et al., 2008; Shao et al., 2018). In addition, various studies have found that anxiety, which is a feature of emotional instability, is related to insula activity (Chua et al., 1999). This finding can be explained by the emotional inflexibility of individuals who score high on emotional instability. Emotional inflexibility is further described by Waugh et al. (2008), who found that low-resilient trait individuals exhibited an earlier rise in insula activity in response to stress induced stimuli, lasting longer than in high-resilient trait individuals, who showed a later rise of neural activity and returned to baseline quicker. Hence, the emotional inflexibility of emotionally instable students might be triggered by stressors related to negative feedback. Simmons et al. (2006) argue that insula activity might be a common feature in individuals with trait anxiety, and suggest that such an activation might be a neuroimaging marker for anxiety proneness. While the findings of the current study contribute to this statement, we have further expanded research into the relationship between personality and psychosocial stress reactions within the insula cortex, as we found that this relationship is far more complex. In line with the proposed H1.2, we found that students who scored high on extraversion at the beginning of 8th grade exhibited significant left insula activation during the stress task. Hence, students who like to engage with other people, enjoy the company and stimulation of others, and are sensitive to social reward respond. We propose that the negative feedback that extraverted students received during the stress task stimulated their left insula cortex, leading them to possibly feel social rejection, social pain, or social punishment as a result of the negative feedback (cf. Ashton et al., 2002). Interestingly, students who reported to be conscientious at the beginning of 8th grade also showed left insula activation during the stress task, which we had not expected or included in our hypothesis. More specifically, left insula activation contributed to the development of students’ conscientiousness from 8th to 9th grade. This finding indicates that students who tend to show orderly behavior, plan, organize, and consistently work to achieve certain goals in 8th grade respond to social stressors neurobiologically. This may be because they feel that they are unable to succeed in the given task, despite their dedication, perseverance, and effort. This experience may be common in students’ school careers and, in turn, reinforces their conscientious behavior over time. In fact, studies indicate that overly pronounced conscientiousness-like constructs, such as perseverance, effort, and perfectionism, may also be related to higher stress levels, as such individuals tend to be concerned about mistakes, feel a discrepancy between expectations and results, and demand too much of themselves (Slaney et al., 2001; Salmela-Aro et al., 2016). The current study contributes to this reasoning by revealing that students who scored high on conscientiousness exhibited greater heart rates during the MIST, which confirms H3.3. Similarly, Lucas et al. (2015) found that conscientious individuals are less likely to give up when failing at a task or losing a game, and that they expend greater persistence and effort, rather than giving up. In order to replicate or reject the findings of the current study, and to investigate how different degrees of conscientiousness may be differentially related to insula activity and heart rate response during induced social stressors, a person-oriented approach may shed light on this question (Bergman and Wangby, 2014).

In contrast to the proposed H1.4, we did not find that insula activity mediated the relationships of agreeableness, extraversion, and emotional instability from the beginning of 8th grade to the end of 9th grade. This may have been due to the small sample size and should be further investigated with larger student groups of different ages. Considering sample size requirements for mediational analyses, Fritz and MacKinnon (2007) indicate that mediating effects could have been detected in the case of our sample if both direct paths had been large (i.e., β ≥ 0.59). However, as both direct paths are small (i.e., β = 0.14), the detection of mediational effects would require more participants.

With regard to the middle temporal gyrus activation during the MIST, we found that conscientious students did not show higher middle temporal gyrus activation, in contrast to H2.1. Furthermore, we expected (H2.2) students who reported high autonomy – which can also be referred to as openness – at the beginning of 8th grade to exhibit lower middle temporal gyrus activation during the MIST, which was not confirmed. However, correlation analysis revealed that right middle temporal gyrus activation in 8th grade was related to the low autonomy of students in 9th grade. Hence, the creative potential and cognitive engagement that is at the core of autonomous students is inhibited in response to socially negative feedback during a complex problem-solving task (Pruessner et al., 2008). Relating these findings to the school context, it is possible that the experience of negative feedback from teachers or peers during a highly demanding task hinders autonomous students from making use of their “natural talent,” and prevents them from being creative, curious, and cognitively engaged with new information. In fact, a meta-analysis revealed that creative performance decreases with increasing stress levels (Byron et al., 2010), while stressed individuals exhibit impairments in attention and have difficulty in monitoring their performance (Mackenzie et al., 2007). In the school context, school-related stress, which may be caused by negative social feedback, is associated with impaired cognitive problem solving (Abdollahi et al., 2018) and inhibits creativity (Pascoe et al., 2019). Furthermore, the results reveal that emotionally instable students at the beginning of 8th grade exhibited greater right middle temporal gyrus activity at the end of 9th grade), which contributes to research by Golde et al. (2021).

H2.3 could not be confirmed, as right middle temporal gyrus activity did not mediate the development of conscientiousness and autonomy over time. Interestingly, students who scored high on agreeableness at the beginning of 8th grade exhibited higher middle temporal gyrus activity at the beginning of 9th grade, which was, in turn, negatively related to their agreeableness at the end of 9th grade. One explanation for this finding could be that the right middle temporal gyrus is positively associated with maladaptive emotion regulation strategies (Golde et al., 2021) such as avoidance, which might be negatively associated with the development of agreeableness. In addition, some studies have found an association between the temporal gyrus and antisocial behavior (see Raine, 2019). However, no indirect effect in this interplay could have been identified. This might be due to the small sample, as both direct effects were small (i.e., β < 0.59) and as such a bigger sample would have been needed to detect significant indirect effect (Fritz and MacKinnon, 2007).

The examination of heart rates during the MIST revealed that emotionally instable students did not show significantly lower heart rates, as H3.1 had expected. However, this might have been due to the study’s limited statistical power, which is a disadvantage of studies with small sample sizes. However, H3.2 was supported by the study’s findings, as highly agreeable students exhibited lower heart rates during the stressful event. As agreeable students tend to engage in quality relationships and tend to be cooperative and helpful to others, so they profit in turn from social relationships that protect them against stressors (Neyer and Lehnart, 2007; Laursen et al., 2010). A wide range of empirical studies underline the importance of social support for healthy development and stress reduction (Papousek et al., 2011; Childs et al., 2014).

To summarize, the results suggest that students with certain personality features (agreeableness, emotional instability, conscientiousness) are likely to exhibit differences in neural activity in different cerebral regions (right and left insula cortex, right middle temporal gyrus). Moreover, neural activity in the left insula cortex partially mediates the longitudinal stability of conscientiousness.

Transferring the results to the school context, in which social feedback from teachers with regard to performance and achievement is part of the daily life of adolescents, the present findings reveal that even short-term negative feedback affects neural processes, and that this might influence personality development. As feedback is a key component of learning (Hattie, 2009), teachers must be sensitized regarding the wide-ranging consequences of their positive and negative feedback. Furthermore, the results indicated the interindividual differences that are caused by certain aspects of personality, which underlines the role of individualized learning methods (i.e., student-centered learning; discovery learning; visible learning) to better foster each student individually.

### Strengths, Limitations, and Future Directions

Some methodical limitations need to be addressed. As is typical in imaging studies, the small sample size limits the statistical power. Future replica studies with larger samples are warranted, which could verify or falsify the present findings. Although the FFM proofed to be a reliable measure, the German FFPI-J used in this study showed certain restrictions in the reliability of autonomy (at T1 and T3), agreeableness (at T1), and extraversion (at T1), which might be due to the age of the participants, as until late adolescence and early adulthood these fluctuations in reliability have been found in other studies as well (Soto et al., 2011; Göllner et al., 2017). Future studies could apply different measures (e.g., EEG, parental or teacher ratings of the students’ big five, etc.) in order to contribute to the present results. Apart from these limitations, this study is one of the few investigations that follows both an interdisciplinary *and* a longitudinal design. It combines fMRI, the heart rate as marker of stressor-induced activation of the autonomic nervous system, and questionnaire data in order to identify associations between brain activity and personality development during adolescence.

## Data Availability Statement

The raw data supporting the conclusions of this article will be made available by the authors, without undue reservation.

## Ethics Statement

The studies involving human participants were reviewed and approved by German Psychological Society. Written informed consent to participate in this study was provided by the participants’ legal guardian/next of kin.

## Author Contributions

DR did the statistical analyses and wrote the method part of the manuscript. FH wrote the discussion part of the manuscript. SK wrote the introduction. SG and TG did the fMRI analyses of the stress task. RL, LR, PP, and AB were mainly involved in the fMRI procedures including conceptualization, data collection, pre-analyses etc. and helped to correcting the manuscript. All the authors contributed to the article and approved the submitted version.

## Conflict of Interest

The authors declare that the research was conducted in the absence of any commercial or financial relationships that could be construed as a potential conflict of interest.

## Publisher’s Note

All claims expressed in this article are solely those of the authors and do not necessarily represent those of their affiliated organizations, or those of the publisher, the editors and the reviewers. Any product that may be evaluated in this article, or claim that may be made by its manufacturer, is not guaranteed or endorsed by the publisher.
