# Supplementary material for: Adolescents’ Personality Development – A Question of Psychosocial Stress
Source: Front Psychol. 2021 Dec 17;12:785610. doi: 10.3389/fpsyg.2021.785610 (PMC8719601; doi:10.3389/fpsyg.2021.785610)
Supplement: Supplementary file 1 [file Data_Sheet_1.docx]

**Supplementary Document A**

**Imaging acquisition.** The experiment was programmed with presentation software (Version 14.9, Neurobehavioral Systems Inc., Albany, CA, USA). A video projector (NEC GT950 NEC Corporation, Itasca, IL, USA, resolution 1024 × 768 pixels) was used to project the experiment onto a screen at the end of the magnet bore. During the fMRI session the participating adolescents look at a mirror at the head coil, which show the video screen. A T1-weighted 3D MPRAGE (magnetization-prepared rapid gradient-echo) sequence with 192 continuous sagittal slices (image matrix = 256 × 256, repetition time [TR] = 1900 ms, echo time [TE] = 2.52 ms, flip angle = 9°, field of view [FOV] = 256 x 256, voxel size = 1 x 1 x 1 mm^3^) was performed. By using axial aligned T2*-weighted gradient echo planar imaging (EPI) sequence sensitive to BOLD contrast (33 transversal slices, image matrix = 64 x 64, *TR* = 2000 ms, *TE* = 30 ms, flip angle = 78°, FoV = 192 x 192, voxel size = 3 x 3 x 3 mm^3^) functional images were collected using (Authors, under review).

**Preprocessing of imaging data.** SPM12 (Statistical Parametric Mapping software package; Wellcome Department of Imaging Neuroscience, London, UK) was used for imaging data analyses. EPIs were adjusted for acquisition time delay and motion artifacts. Afterwards - as implemented in SPM12 – they were transformed into the stereotactic normalized standard space of the Montreal Neuroimaging Institute using the unified segmentation algorithm. In a last step, EPIs were resampled (voxel size = 3 × 3 × 3 mm^3^) and smoothed with a 3D Gaussian kernel of 7 mm full-width-at-half-maximum (Authors, under review).

**Supplementary Table A:** One sample t-test to compare high and low stress conditions (t-contrast: high stress > low stress)

| **Brain Region** | **H** | **Cluster size** (Voxels) | **T**  (Peak) | **P**  (FWE) | **MNI coordinates**  x y z | | |
| --- | --- | --- | --- | --- | --- | --- | --- |
| ACC | L | 400 | 8.71 | <.0001 | -3 | 35 | 19 |
| ACC | R |  | 8.20 | <.0001 | 0 | 26 | 16 |
| ACC | L |  | 6.92 | .0001 | -3 | 29 | 1 |
| Insula Lobe | R | 107 | 8.51 | <.0001 | 30 | 17 | -11 |
| Insula Lobe | R |  | 7.69 | .006 | 39 | 14 | -8 |
| Insula Lobe | L | 41 | 6.58 | .002 | -27 | 17 | -8 |
| Insula Lobe | L |  | 5.61 | .024 | -30 | 11 | -17 |
| Middle Temporal Gyrus | R | 37 | 7.70 | .006 | 60 | -13 | -23 |
| Angular Gyrus | L | 22 | 6.05 | .007 | -54 | -58 | 34 |
| Middle Temporal Gyrus (anterior) / Temporal Pole | R | 16 | 6.60 | .002 | 54 | 5 | -29 |
| Subcallosal Gyrus | R | 13 | 6.50 | .002 | 6 | 23 | -14 |

*Note.* Peak voxel coordinates and t-values at *p* < .05; FWE = Family-wise Error (corrected across the whole brain), cluster size ≥ 5 voxels; H = hemisphere; L = left, R = right, ACC = Anterior Cingulate Cortex; MNI = Montreal Neurological Institute (see Authors, 2021).

**Supplementary Document B**

INPUT INSTRUCTIONS MPLUS

TITLE: stress task;

DATA: FILE = "stress_task.dat";

VARIABLE:

NAMES =

RIns_2

RMidTemp_4

LIns_5

aSOC

aAGREE

aCONS

aEMOST

aauto

bsoc

bAGREE

bCONS

bEMOST

bAUTO

hHR;

MISSING=ALL(-99);

USEVARIABLES =

RIns_2

RMidTemp_4

LIns_5

aSOC

aAGREE

aCONS

aEMOST

aauto

bsoc

bAGREE

bCONS

bEMOST

bAUTO

hHR;

ANALYSIS:

ESTIMATOR = MLR;

MODEL:

bsoc on

asoc;

bAGREE on

aagree;

bCONS on

acons;

bEMOST on

aemost;

bauto on

aauto;

bsoc on

RIns_2

RMidTemp_4

LIns_5

;

bagree on

RIns_2

RMidTemp_4

LIns_5

;

bcons on

RIns_2

RMidTemp_4

LIns_5

;

bemost on

RIns_2

RMidTemp_4

LIns_5

;

bauto on

RIns_2

RMidTemp_4

LIns_5

;

ASOC AAGREE ACONS AEMOST AAUTO WITH

hHR;

BSOC BAGREE BCONS BEMOST BAUTO WITH

hHR;

RIns_2 with

RMidTemp_4

LIns_5 hhr;

RMidTemp_4 with

LIns_5 hhr;

LIns_5 with hhr;

RIns_2 RMidTemp_4 LIns_5 ON

aSOC

aAGREE

aCONS

aEMOST

aAUTO

;

aAUTO WITH

aAGREE

aCONS

aEMOST

aSOC

;

aAGREE WITH

aCONS

aEMOST

aSOC

;

aCONS WITH

aEMOST

aSOC

;

aEMOST WITH

aSOC;

RIns_2 WITH

RMidTemp_4

LIns_5;

RMidTemp_4 WITH

LIns_5;

model indirect:

bsoc ind Rins_2 asoc;

bsoc ind Rmidtemp_4 asoc;

bsoc ind Lins_5 asoc;

bcons ind Rins_2 acons;

bcons ind Rmidtemp_4 acons;

bcons ind Lins_5 acons;

bagree ind Rins_2 aagree;

bagree ind Rmidtemp_4 aagree;

bagree ind Lins_5 aagree;

bemost ind Rins_2 aemost;

bemost ind Rmidtemp_4 aemost;

bemost ind Lins_5 aemost;

bauto ind Rins_2 aauto;

bauto ind Rmidtemp_4 aauto;

bauto ind Lins_5 aauto;

OUTPUT:

sampstat stdyx modindices cinterval;
